# Integrative molecular diagnostics for HPV-driven cervical carcinogenesis: a translational review of mechanisms and multimodal risk stratification

**DOI:** 10.3389/fonc.2026.1803942

**Published:** 2026-03-23

**Authors:** Deema Ibrahim Fallatah, Hafeez Aderinsayo Adekola

**Affiliations:** 1 Department of Medical Laboratory, College of Applied Medical Sciences, Prince Sattam bin Abdulaziz University, Alkharj, Saudi Arabia; 2 Department of Molecular Biology and Biotechnology, Nigerian Institute of Medical Research, Lagos, Nigeria

**Keywords:** cervical cancer, genomic instability, human papillomavirus (HPV), molecular diagnostics, risk stratification

## Abstract

Despite effective prophylactic vaccines, high-risk human papillomavirus (HPV) infection remains a leading cause of cervical cancer, particularly in regions with limited screening access. Conventional methods, including cytology and HPV DNA testing, lack specificity for identifying lesions at high risk of progression, resulting in overtreatment. This narrative translational review synthesizes current evidence on integrating complementary molecular modalities to improve risk stratification along the biological continuum from HPV infection to malignancy. Persistent high-risk HPV drives carcinogenesis via viral oncoprotein activity, host DNA damage response (DDR) dysregulation, and epigenetic remodeling, yet no single biomarker captures this complexity. Quantitative PCR sensitively detects viral DNA load but not transforming activity; immunocytochemistry for p16INK4a/Ki-67 reflects oncogenic pathway disruption; gene expression and methylation profiling reveal downstream transcriptional changes; and emerging DDR assays (e.g., γH2AX) indicate upstream genomic stress. Multi-omics studies suggest progression risk is best inferred from co-occurring viral activity, host stress responses, and phenotypic dysregulation. A multimodal approach combining viral detection, protein-level transformation markers, and selected molecular signatures, provides a biologically grounded framework to distinguish transient infection from high-risk precancer. Literature was identified through targeted searches of PubMed, Scopus, and Web of Science, emphasizing peer-reviewed studies, meta-analyses, translational investigations, and clinically validated diagnostic platforms published in English. This review proposes a structured, integrative model for cervical cancer risk assessment, offering a tiered, context-appropriate strategy that correlates diagnostic modalities with stages of HPV-mediated transformation. This framework aims to enhance clinical precision, prioritize high-risk individuals, and reduce overtreatment.

## 1 Introduction

Human papillomavirus (HPV) is one of the most common sexually transmitted infections, comprising over 200 identified types that are classified as low-risk or high-risk based on oncogenic potential (1). High-risk HPV types are etiologically linked to cervical cancer, accounting for approximately 70% of cases globally (2). Cervical cancer remains a major public health burden, with more than 600,000 new cases and over 300,000 deaths reported annually worldwide (3). Although prophylactic vaccination has significantly reduced the incidence of high-risk HPV infection in vaccinated populations, barriers such as vaccine hesitancy, cost, and limited healthcare infrastructure continue to impede equitable global implementation, particularly in low- and middle-income countries (4, 5).

### 1.1 Core molecular mechanisms of HPV-mediated oncogenesis

**Figure 1:**
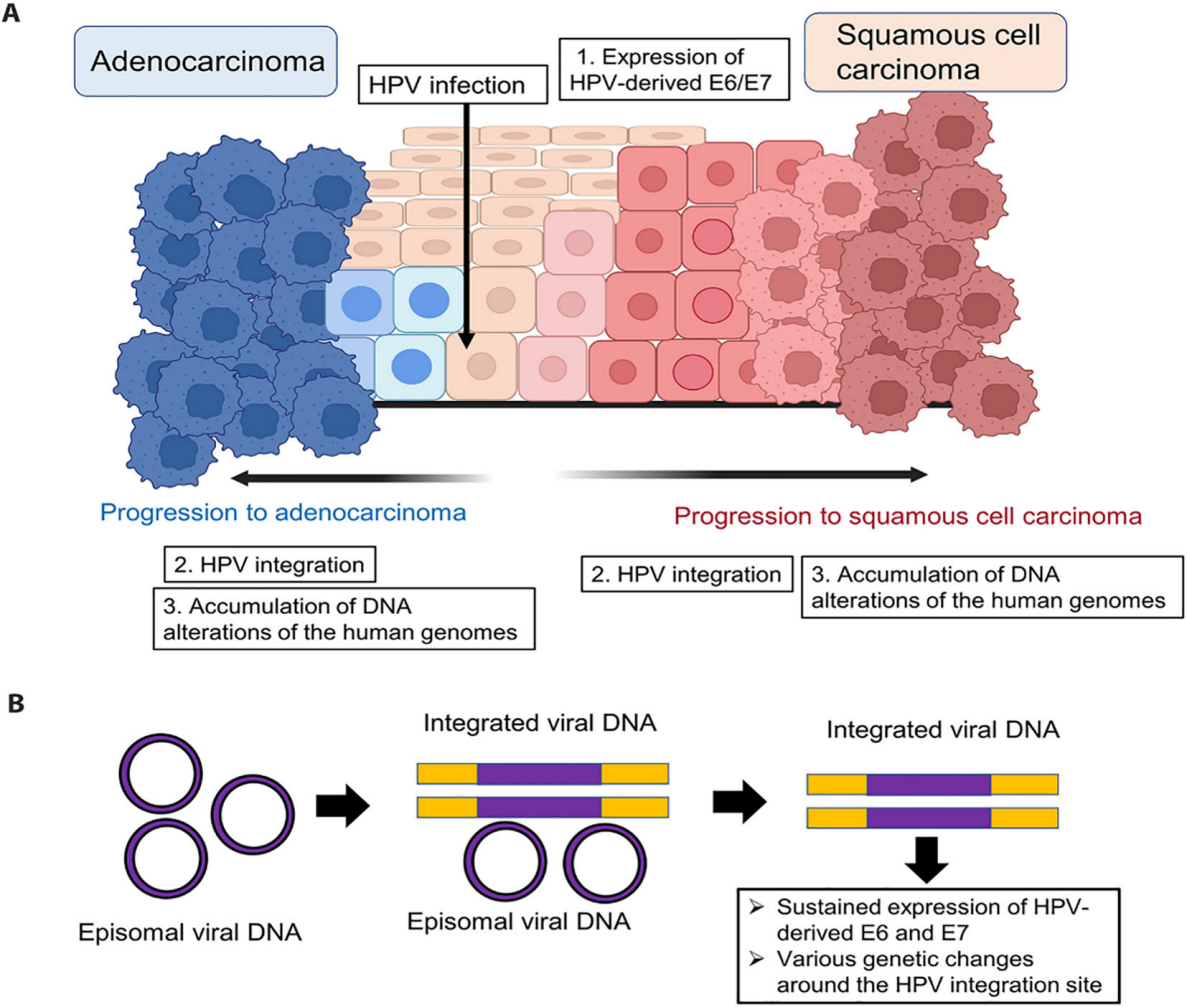
Tumorigenesis mechanisms by HPV Source: Kusakabe et al., (6). **(A)** Tumorigenesis mechanism of uterine cervical carcinoma. HPV infects basal cervical cells, driving tumorigenesis over years. Squamous cell carcinoma progresses stepwise from cervical intraepithelial neoplasia (CIN) precursors. **(B)** Changes in HPV genomic status during cervical carcinogenesis. HPV genome integration into the host genome occurs during persistent infection. This integration enables sustained E6/E7 expression and induces genetic alterations, including oncogene amplification, chromosomal rearrangements, and genomic instability at integration sites.

High-risk HPV–driven carcinogenesis is primarily mediated by sustained expression of the viral oncoproteins E6 and E7 following infection of basal epithelial cells (**Figure 1**) (7). The E6 oncoprotein promotes ubiquitin-mediated degradation of the tumor suppressor p53, thereby impairing DNA damage responses and apoptosis. Concurrently, E7 functionally inactivates the retinoblastoma protein (pRb), leading to deregulated E2F transcription factor activity and uncontrolled cell cycle progression. Persistent disruption of these tumor suppressor pathways induces replication stress, chromosomal instability, and accumulation of genetic alterations, establishing a permissive cellular environment for malignant transformation (7).

Beyond direct oncogene activity, HPV infection itself contributes to genomic instability. During viral replication, abnormal DNA structures including stalled replication forks and single- or double-strand breaks can overwhelm host DNA repair mechanisms, increasing reliance on error-prone repair pathways such as non-homologous end joining (NHEJ) (8–10). Viral–host interactions involving regulatory proteins such as HPV16 E2 and cellular factors including TOPBP1 further modulate DNA damage responses, highlighting the complex interplay between viral persistence and host genome integrity (11, 12). While viral DNA integration is a frequent event in advanced disease, particularly with HPV-16 and HPV-18 (**Table 1**), it is increasingly recognized as a consequence of accumulated genomic stress rather than an obligatory initiating step (13).

**Table 1:** Molecular characteristics of selected HPV genotypes.

HPV Genotype	Key Oncoproteins	Known molecular actions	p16/Ki-67 Overexpression
HPV-16	E6, E7	E6 degrades p53, E7 inactivates Rb	Yes
HPV-18	E6, E7	Strong p53 suppression, associated with glandular lesions	Yes
HPV-31	E6, E7	Moderate oncogenic potential, integrates into host genome	Yes
HPV-33	E6, E7	Alters cell cycle regulators	Yes
HPV-6	E6, E7	Weak p53 binding; rarely integrates into host genome	No
HPV-11	E6, E7	Similar to HPV-6, low oncogenicity	No

Current cervical cancer screening approaches, including cytology (Pap smear) and HPV DNA testing, have substantially reduced disease burden but remain limited in their ability to accurately discriminate between transient HPV infections and lesions with a high likelihood of progression. Cytology-based screening suffers from variable sensitivity and inter-observer variability, while HPV DNA testing lacks specificity for clinically significant disease, often resulting in overdiagnosis and unnecessary interventions (14, 15). These limitations are particularly consequential in resource-limited settings, where efficient allocation of diagnostic and clinical resources is critical.

Accordingly, there is a growing need for molecular markers that can more accurately reflect biological progression risk rather than mere viral presence. Multimodal strategies integrating quantitative PCR (qPCR), gene expression profiling, and immunocytochemical markers offer a promising avenue for improving risk stratification by capturing complementary dimensions of HPV-driven cellular transformation. Such approaches have the potential to reduce overtreatment, prioritize high-risk individuals, and enhance the clinical precision of cervical cancer prevention programs (16, 17)This article is presented as a narrative translational review that synthesizes current evidence on HPV-driven cervical carcinogenesis, with emphasis on molecular mechanisms and their diagnostic and therapeutic implications. Rather than proposing a clinically validated algorithm, this review integrates findings from molecular biology, pathology, and translational oncology literature to conceptually evaluate how diverse molecular modalities complement one another across the spectrum of HPV infection and disease progression. By emphasizing biological plausibility, mechanistic coherence, and clinical relevance, particularly in low- and middle-income settings. This review aims to provide a structured framework for interpreting existing biomarkers and identifying opportunities to improve risk stratification and patient triage using currently available evidence.

### 1.2 Literature search strategy and review design

This article is structured as a narrative translational review rather than a systematic or scoping review. A targeted literature search was conducted using PubMed, Scopus, and Web of Science databases. Search terms included combinations of: “HPV carcinogenesis,” “E6/E7,” “DNA damage response,” “p16/Ki-67,” “HPV methylation biomarkers,” “HPV viral load,” “multi-omics cervical cancer,” and “molecular triage.” Studies published in English were considered. Priority was given to peer-reviewed original research, meta-analyses, large cohort studies, randomized screening trials, and translational investigations published within the last 10–15 years, while seminal mechanistic studies were included irrespective of publication date. Inclusion criteria focused on studies evaluating molecular mechanisms of HPV-driven transformation, diagnostic biomarker performance, and translational applicability in clinical or screening contexts. Preclinical DDR-focused studies were included when directly relevant to mechanistic interpretation. Case reports, non-peer-reviewed articles, and studies lacking methodological clarity were excluded. As a narrative review, formal systematic screening, PRISMA flow diagrams, and quantitative meta-analysis were not performed. The objective was conceptual integration and mechanistic synthesis rather than exhaustive evidence aggregation.

## 2 Current understanding of HPV driven pathogenesis

The majority of HPV infections are transient and are cleared by the host immune system within one to two years; however, persistent infection with high-risk HPV types substantially increases the risk of cervical carcinogenesis (2). Viral persistence is influenced by host and environmental factors, including immune suppression, smoking, prolonged oral contraceptive use, co-infection with other sexually transmitted pathogens (e.g., *Chlamydia trachomatis*), early sexual debut, multiple sexual partners, and limited access to healthcare services (18–21). These factors impair viral clearance and facilitate long-term viral maintenance within the cervical epithelium.

### 2.1 Episomal persistence and early replication stress

In early stages of infection, high-risk HPV genomes are typically maintained in an episomal state. Even in the absence of viral integration, episomal HPV replication imposes substantial replication stress on host cells. Even in the absence of integration, episomal replication amplifies the replication stress and DNA damage response activation described in Section 1.1, promoting early genomic instability. (22, 23).

### 2.2 Viral integration and escalation of genomic instability

As genomic instability accumulates, viral DNA integration into the host genome becomes more likely, particularly in advanced precancerous lesions and invasive carcinomas (13). Integration events frequently disrupt viral regulatory regions, resulting in deregulated expression of E6 and E7 and amplification of their downstream oncogenic effects. Importantly, integration also alters host genomic architecture, leading to chromosomal rearrangements, copy number variations, and dysregulated expression of genes proximal to integration sites (24). Beyond local effects, viral integration can induce broader epigenomic alterations, including changes in chromatin accessibility and DNA methylation patterns, which further reinforce malignant phenotypes (25).

### 2.3 Epigenetic remodeling and pathway dysregulation

HPV-associated carcinogenesis is accompanied by extensive epigenetic remodeling; however, these changes are context dependent. In HPV-driven cervical lesions, the cyclin-dependent kinase inhibitor p16^INK4a^ is characteristically overexpressed, reflecting functional inactivation of the retinoblastoma pathway by HPV E7 rather than promoter silencing. Loss of Rb-mediated repression leads to compensatory upregulation of p16^INK4a^, underpinning its utility as a surrogate marker of transforming high-risk HPV infection. In contrast, promoter hypermethylation more commonly affects other tumor suppressor genes involved in cell cycle regulation, DNA repair, and apoptosis, such as *RB1*, *TP53*, and *CDH1*, particularly in advanced disease stages or HPV-independent tumors. Reports of p16^INK4a^ promoter methylation are largely confined to HPV-negative or non-cervical malignancies and do not explain its diagnostic value in HPV-associated cervical disease.

### 2.4 Immune evasion and tumor evolution

Persistent HPV infection is further facilitated by viral strategies that attenuate host immune surveillance. HPV limits viral protein expression and disrupts interferon signaling pathways, suppressing the induction of interferon-stimulated genes and reducing antigen presentation through downregulation of major histocompatibility complex molecules (26). Concurrent modulation of cytokine profiles, characterized by reduced pro-inflammatory and enhanced immunosuppressive signaling, creates a permissive microenvironment for viral persistence, clonal selection, and tumor evolution (27).

### 2.5 Histopathological progression and biomarker dynamics

Cervical carcinogenesis follows a well-defined histopathological continuum, progressing from low-grade squamous intraepithelial lesions (LSIL) to high-grade squamous intraepithelial lesions (HSIL), and ultimately to invasive carcinoma if left untreated (28). This progression is accompanied by increasing genomic instability and distinct biomarker changes. HSILs and invasive cancers exhibit elevated expression of proliferation and transformation markers, including Ki-67 and p16^INK4a^, reflecting deregulated cell cycle control and accumulating molecular damage (29, 30). Quantitative measures such as HPV DNA load and viral gene expression profiles have emerged as potential indicators of progression risk, with higher viral loads correlating with increased likelihood of transition from cervical intraepithelial neoplasia to invasive disease (31–33).

## 3 Integrative molecular approaches for HPV-driven cancer

Quantitative PCR (qPCR) is a cornerstone molecular technique for the detection and quantification of human papillomavirus (HPV) DNA in cervical samples. By using sequence-specific primers and fluorescent probes, qPCR enables sensitive identification of HPV genotypes, including discrimination between high-risk and low-risk strains, and allows quantification of viral load (34). Viral load measurements have been associated with disease persistence and increased risk of cervical intraepithelial neoplasia and invasive cancer (31, 35). Compared with cytology-based methods, qPCR offers high analytical sensitivity and reproducibility, facilitating early detection of HPV infection and monitoring of viral dynamics over time (36, 37). However, while qPCR provides valuable information on viral presence and burden, it does not directly capture downstream biological consequences such as host-cell transformation, genomic instability, or disruption of DNA repair pathways.

Gene expression profiling provides a complementary layer of information by characterizing transcriptional alterations induced by HPV infection and progression toward malignancy. Dysregulated expression of genes involved in cell cycle control, apoptosis, immune modulation, and genomic stability has been consistently observed in HPV-associated cervical lesions and cancers (38). High-throughput platforms, including microarray analysis and RNA sequencing, have enabled comprehensive assessment of these transcriptional changes, facilitating tumor subclassification and identification of candidate prognostic markers (39). In particular, altered expression of genes involved in homologous recombination and non-homologous end joining pathways reflects host-cell adaptation to sustained replication stress and DNA damage induced by HPV persistence (40). While gene expression profiling remains largely confined to research and specialized clinical settings, integration of transcriptional data with clinical metadata may inform risk stratification and therapeutic decision-making in selected contexts.

Immunocytochemistry (ICC) represents a more clinically established modality by enabling visualization of protein-level changes associated with HPV-driven transformation. Markers such as p16^INK4a^ and Ki-67 are widely used to assess deregulated cell cycle activity and proliferative status in cervical epithelial cells (41, 42). p16INK4a and Ki-67 dual staining functions as a surrogate indicator of transforming infection. Combined assessment of these markers improves lesion classification, supports differentiation between benign and precancerous changes, and aids clinical decision-making, particularly in histopathological triage (42). Compared with molecular assays requiring advanced infrastructure, ICC offers greater feasibility for routine diagnostic workflows and remains especially relevant in resource-constrained settings when standardized protocols are applied.

HPV-driven carcinogenesis is also characterized by extensive epigenetic remodeling, including DNA methylation, histone modifications, and altered expression of non-coding RNAs (43). Aberrant DNA methylation patterns, such as hypermethylation of host tumor suppressor genes and locus-specific changes including *PAX1*, have been proposed as potential biomarkers for early detection and risk stratification (44, 45). Histone modifications mediated by interactions between HPV oncoproteins and host chromatin-modifying enzymes further contribute to transcriptional dysregulation and immune evasion (46–48). Additionally, altered expression of microRNAs and long non-coding RNAs—such as upregulation of miR-21 and dysregulation of MALAT1 and HOTAIR has been associated with tumor progression, invasion, and metastasis in HPV-positive cervical cancer (39, 49, 50). Although epigenetic markers hold promise for biomarker development and therapeutic targeting, most remain under investigation and require further validation before routine clinical implementation (51).

To enhance diagnostic performance, increasing attention has been given to multi-marker panels that integrate viral, host, and protein-level indicators of disease progression. Panels incorporating HPV DNA or mRNA testing alongside immunocytochemical markers such as p16^INK4a^, Ki-67, and MCM2 have demonstrated improved sensitivity and specificity for identifying high-grade lesions while reducing overtreatment (52, 53). These approaches offer a pragmatic balance between biological depth and clinical feasibility and may be adaptable to automated platforms suitable for large-scale screening initiatives (54, 55). Advanced systems biology approaches including proximity ligation assays, network-based modeling, and high-resolution imaging, have further elucidated the spatial and temporal dynamics of HPV–host interactions, particularly in relation to DNA repair disruption and immune modulation (27, 56, 57).

Translational limitations of DDR-based assays must therefore be explicitly acknowledged. While γH2AX detection, comet assays, proximity ligation assays, and high-resolution imaging have substantially advanced understanding of HPV-induced replication stress and genomic instability, they lack standardized thresholds, prospective clinical validation, and scalability required for population-level screening. These constraints are particularly pronounced in low- and middle-income countries, where cost, infrastructure, and workforce capacity are critical considerations. Consequently, DDR assays are best regarded as exploratory or adjunctive tools that inform mechanistic models of disease progression or identify therapeutic vulnerabilities such as sensitivity to DNA-damaging agents, rather than as standalone diagnostic tests.

## 4 Precision diagnostics: integrating multi-omics approaches

Integrated multi-omics analyses have refined molecular classification of HPV-driven cervical cancer. Genomic analyses reveal genetic alterations, transcriptomics captures dysregulated gene expression, proteomics reflects protein-level changes, and epigenomics provides insight into regulatory modifications. While multi-omics studies also capture host genomic instability signatures (8, 58).

Large-scale studies, including TCGA (2017) (59), Chakravarthy et al. (60) Li et al. (61) Liu et al. (62), and Qiu et al. (63), consistently highlight recurrent molecular features of HPV-driven cervical cancer. These include dysregulation of cell cycle control (notably E2F and p53/Rb pathways), activation of DNA damage response pathways, immune evasion, chromatin remodeling, and metabolic reprogramming. Proteomic and phosphoproteomic analyses reinforce these findings, linking productive HPV integration to increased E6/E7 expression, tumor aggressiveness, and altered immune signaling. Multi-omics data also identify potential biomarkers, such as TMAO metabolites, NOD1 dysregulation, and microbiome shifts, that may inform non-invasive or adjunctive monitoring strategies (60–64).

Despite methodological diversity, multi-omics studies converge on a set of recurring molecular hallmarks that define high-risk HPV-driven lesions: persistent viral activity, host DNA repair disruption, immune microenvironment remodeling, and epigenetic reprogramming. Clinically, while comprehensive multi-omics profiling is not yet feasible for routine screening, these insights inform the rational selection of surrogate markers. For instance, sustained E6/E7 activity and DNA repair perturbations can be captured by p16^INK4a^ and Ki-67 immunocytochemistry, while methylation and viral load dynamics provide complementary information. Multi-omics studies function as a conceptual scaffold for designing multimodal risk stratification approaches, guiding early detection and patient triage in both research and clinical settings.

Despite biological richness, multi-omics approaches remain largely discovery-stage tools. Current limitations include cost, analytic complexity, lack of standardization, and absence of prospective screening-level validation. Their present value lies in mechanistic elucidation and biomarker discovery rather than clinical implementation.

### 4.1 Proposed tiered integrative workflow for clinical application

For operational clarity, progression through tiers should be triggered by predefined positivity criteria. For example, primary hrHPV positivity (PCR-based detection of one or more high-risk genotypes) would prompt reflex triage using p16/Ki-67 dual staining or methylation analysis. A positive triage result, defined according to manufacturer-recommended cut-offs or validated laboratory thresholds, would warrant referral for colposcopy. Negative triage results could enter repeat testing at 12 months, depending on risk stratification and age.

Although the modalities discussed in this review are presented as complementary, several reflect convergent biological processes within the HPV-driven transformation cascade. For example, DNA methylation alterations and p16INK4a/Ki-67 dual immunocytochemistry both arise downstream of sustained E6/E7 oncogene activity and retinoblastoma pathway disruption. These biomarkers may therefore capture overlapping transformation states rather than fully independent molecular events. Recognizing this mechanistic convergence is essential when designing multimodal or tiered algorithms to ensure that each additional layer provides incremental diagnostic value rather than redundant stratification.

**Figure 2:**
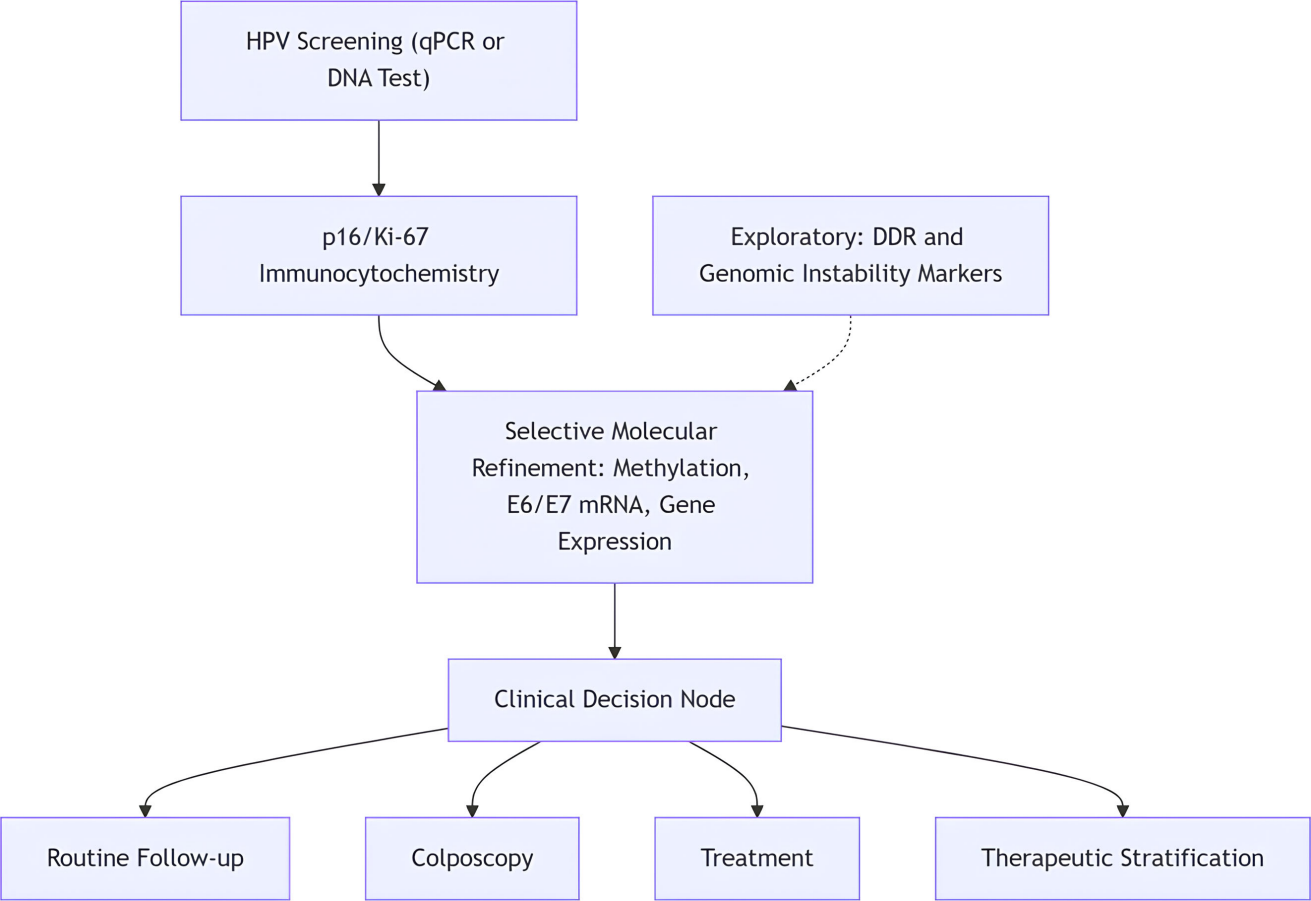
Proposed tiered integrative molecular workflow for HPV-driven cervical cancer risk stratification.

To enhance translational applicability, the conceptual integrative model described in this review can be operationalized as a tiered diagnostic workflow aligned with resource availability and biological risk assessment (**Figure 2**).

Tier 1: primary screening (viral detection and genotyping): High-risk HPV detection using qPCR or validated HPV DNA assays serves as the initial screening step. Viral load quantification may provide additional risk stratification, particularly in persistent infections.

Tier 2: transformation assessment (protein-level biomarkers): Samples positive for high-risk HPV undergo immunocytochemical evaluation for p16INK4a and Ki-67 co-expression. Dual positivity indicates functional disruption of the Rb pathway and increased likelihood of transforming infection.

Tier 3: molecular risk refinement (selective advanced testing): In cases with persistent HPV infection, discordant cytology, or clinical uncertainty, adjunct molecular testing may be considered. These may include:

- Host or viral DNA methylation markers (e.g., PAX1, FAM19A4/miR124-2).
- HPV E6/E7 mRNA detection.
- Gene expression profiling in specialized settings.

Exploratory/research layer: genomic stress and DDR profiling: Markers of replication stress or DNA damage response disruption (e.g., γH2AX, ATM pathway alterations) may provide mechanistic insight and inform therapeutic vulnerability but are not currently recommended for routine screening.

The tiered framework prioritizes scalability and feasibility, allowing stepwise intensification of diagnostic evaluation based on biological risk. This approach supports precision triage while minimizing overtreatment, particularly in low- and middle-income settings.

While precise numerical thresholds may vary by assay and population, implementation of the integrative model requires locally validated decision cut-offs. These may include quantitative cycle threshold (Ct) values for hrHPV viral load, percentage methylation indices above predefined discriminatory levels, or dual-stain positivity based on established cytomorphologic criteria. The workflow is therefore intended as a framework that incorporates evidence-based assay thresholds rather than replacing them. Prospective validation studies would be necessary to determine optimal cut-offs that balance sensitivity for CIN2+ detection with acceptable specificity.

## 5 Therapeutic implications and future directions

### 5.1 Biomarker-informed therapy selection

Molecular diagnostics provide critical information that can guide therapeutic decisions in HPV-driven cervical cancer. For instance, immunocytochemistry (ICC) markers such as p16^INK4a^ and Ki-67, alongside viral load quantification, can identify patients with transforming HPV infections who are more likely to benefit from immunotherapy or combination treatments (65). Profiling gene expression and pathway activity (e.g., PI3K/AKT, MAPK) allows clinicians to identify dysregulated signaling networks, informing the selection of targeted inhibitors or combination strategies (66, 67). Similarly, assessment of DNA damage response (DDR) status, for example, alterations in ATM, BRCA1/2, or TOPBP1-mediated repair pathways, can pinpoint tumors with repair deficiencies that may be more sensitive to DDR-targeted therapies or radiosensitizers (8–10).

### 5.2 Clinical and translational applications

Evidence from clinical trials indicates that immune checkpoint inhibitors (ICIs) and therapeutic vaccines targeting E6/E7 oncoproteins can improve outcomes in HPV-driven cancer (68,69). Molecular diagnostics enhance patient selection by identifying those with persistent viral activity, elevated viral load, or high-risk molecular signatures, thereby increasing the probability of therapeutic benefit. Similarly, combination therapies can be rationally designed by leveraging molecular insights; for example, tumors with co-activated PI3K/AKT and MAPK pathways may require dual inhibition to overcome treatment resistance (66, 67). DDR profiling can further inform the use of radiosensitizers or PARP inhibitors in patients with compromised DNA repair.

### 5.3 Future directions: experimental therapeutic technologies

Emerging therapies, such as CRISPR-based gene editing and CAR-T cell therapy, represent promising experimental strategies for HPV-related cancers (70–72). These approaches aim to disrupt viral oncogene expression or direct immune responses against HPV-infected cells. At present, they remain preclinical or early-phase investigational tools. Molecular diagnostics will be essential for their eventual clinical application, enabling personalized targeting based on HPV genotype, viral load, and oncogene activity. Future research should continue to evaluate these approaches within the context of established biomarker-informed patient stratification.

## 6 Challenges and considerations

Implementing precision medicine encounters several challenges related to cost, accessibility, and technical limitations (73). Advanced molecular diagnostic techniques necessitate sophisticated equipment and specialized personnel, which can be difficult to obtain in low- and middle-income countries. Furthermore, the requirement for ongoing training and education adds additional pressure on already limited resources, resulting in a knowledge gap among healthcare providers (74). Accessibility to these advanced diagnostics is particularly restricted in rural and underserved areas (73). Technical limitations may also introduce variability in test results, complicating the consistent use of molecular diagnostics in various healthcare settings (75). Integrating quantitative PCR (qPCR) and gene expression profiling into clinical practice presents challenges, including the need for standardized protocols, data interpretation, and logistical considerations. Inconsistent sample collection, processing, and analysis can result in unreliable outcomes, making it challenging for clinicians to trust these diagnostic tests. In addition to these barriers, assays that monitor DNA repair disruption or measure replication-associated damage, though promising, remain technically demanding and costly (76). Their implementation in low-resource settings is limited not only by infrastructure but also by the lack of simplified protocols suitable for routine use. Addressing this challenge requires further research to adapt and validate DNA damage response assays in point-of-care formats, making them more feasible in rural or underserved regions. To enhance the reliability of molecular diagnostics, it is essential to establish standardized procedures and develop specialized laboratory infrastructure (73, 77). Moreover, incorporating precision diagnostics into therapeutic decision-making necessitates careful consideration of regulatory and logistical frameworks to ensure safety, efficacy, and adherence to ethical standards. Regulatory agencies such as the FDA play vital roles in establishing guidelines for new diagnostic tests.

### 6.1 Tiered implementation framework for resource-constrained settings

To facilitate pragmatic adoption across heterogeneous health systems, a tiered implementation model may better align diagnostic sophistication with infrastructure capacity.

Tier 1: minimum package (population-level screening focus): Designed for primary care and decentralized settings, this tier prioritizes high-sensitivity HPV DNA testing, including self-sampling strategies where feasible. Visual triage methods or simplified molecular triage (where available) may be incorporated. Emphasis should be placed on affordability, supply chain stability, and minimal laboratory complexity.

Tier 2: intermediate package (structured molecular triage): Applicable to district-level laboratories with basic molecular capability, this tier incorporates partial HPV genotyping and/or validated triage biomarkers such as p16/Ki-67 dual staining. Quality assurance systems, trained cytotechnologists, and structured referral pathways are essential components.

Tier 3: advanced package (centralized precision risk stratification): Reserved for tertiary or national reference centers, this level may integrate expanded molecular profiling, host methylation assays, or research-stage multi-marker panels for risk refinement. These approaches require robust bioinformatics support, standardized protocols, and sustainable financing models.

## 7 Critical appraisal of diagnostic performance and evidence quality

Reported performance metrics are derived from published randomized screening trials, multicenter validation studies, or meta-analyses using histologically confirmed CIN2+ or CIN3+ as reference standards where specified. Given methodological heterogeneity across platforms and study designs, ranges are presented rather than pooled estimates. Although multiple molecular and immunocytochemical modalities demonstrate biological plausibility across the continuum of HPV-mediated transformation, the strength of supporting evidence varies substantially across platforms.

HPV DNA testing and viral load: High-risk HPV DNA testing is supported by large randomized trials and population-based screening programs, demonstrating high sensitivity (>90%) for CIN2+ detection but limited specificity due to transient infections (78). Viral load quantification shows heterogeneous predictive value across studies, and thresholds remain non-standardized, limiting routine clinical implementation (79).

p16/Ki-67 dual immunocytochemistry: Dual staining exhibits improved specificity compared with HPV DNA testing alone, with reported sensitivities typically ranging from 75–80% and superior triage performance in HPV-positive women (80). Evidence is supported by multicenter validation studies; however, inter-observer variability and cost considerations remain relevant in low-resource settings (81).

DNA methylation markers: Host and viral methylation assays demonstrate promising specificity for high-grade lesions, often exceeding cytology-based triage. Nonetheless, heterogeneity in gene targets, assay platforms, and cutoffs limits cross-study comparability. Large-scale prospective validation and cost-effectiveness analyses are still emerging (82).

E6/E7 mRNA detection: mRNA-based assays increase specificity by targeting transcriptionally active infection, yet sensitivity may be modestly lower than or equivalent to DNA-based assays. Clinical adoption varies geographically, and head-to-head comparisons across platforms remain limited (83).

Gene expression and multi-omics approaches: Transcriptomic and integrated multi-omics analyses provide mechanistic insight and potential prognostic refinement. However, current evidence is largely derived from retrospective or discovery cohorts, with limited external validation. Standardization, reproducibility, bioinformatic complexity, and cost remain substantial barriers to clinical translation (84).

DNA damage response (DDR) and genomic instability markers: DDR-associated biomarkers (e.g., γH2AX, ATM/ATR pathway alterations) are mechanistically compelling but remain investigational. Most supporting data originate from preclinical or small translational studies. Their utility in routine screening or triage is currently unsupported by large prospective trials (85, 86).

## 8 Conclusion

This review synthesizes mechanistic and diagnostic evidence to propose a tiered integrative framework for HPV-related risk stratification. This review highlights that quantitative PCR, gene expression profiling, and immunocytochemistry provide complementary insights into viral persistence, host response, and cellular transformation, forming a conceptual framework for risk stratification. Integrating these molecular and protein-level markers enhances identification of high-risk lesions and supports informed triage decisions, particularly in resource-limited settings. Moreover, biomarker-informed approaches can guide personalized therapeutic strategies, including immunotherapy, targeted pathway inhibition, and DNA damage response–directed interventions. Implementing tiered diagnostic workflows grounded in a structured integrative algorithm can facilitate stepwise risk stratification from viral detection to transformation assessment and molecular refinement. Such a framework provides clinicians with a pragmatic decision-support structure while preserving biological coherence across disease stages.
